# Widely Targeted Metabolomics Method Reveals Differences in Volatile and Nonvolatile Metabolites in Three Different Varieties of Raw Peanut by GC–MS and HPLC–MS

**DOI:** 10.3390/molecules29225230

**Published:** 2024-11-05

**Authors:** Jiantao Fu, Yuxing An, Dao Yao, Lijun Chen, Liwen Zhou, Dachun Shen, Sixing Dai, Yinglin Lu, Donglei Sun

**Affiliations:** 1 Institute of Nanfan & Seed Industry, Guangdong Academy of Sciences, Guangzhou 510316, China; fjtxmy@126.com (J.F.);; 2 Guangdong Province Pesticide-Fertilizer Technology Research Center, Guangzhou 510316, China; 3 Heyuan Provincial Academy of Sciences Research Institute, Guangdong Academy of Sciences, Heyuan 517001, China

**Keywords:** raw peanut, variety, widely targeted metabolome, volatile metabolites, nonvolatile metabolites

## Abstract

The aim of the present study was to comprehensively analyze and identify the metabolites of different varieties of raw peanut, as well as provide a reference for the utilization of different varieties of peanuts. In this study, three varieties of peanuts, namely ZKH1H, ZKH13H, and CFD, were investigated via ultrahigh-performance liquid chromatography (UPLC) and widely targeted metabolomics methods based on tandem mass spectrometry (MS) and solid-phase microextraction-gas chromatography–mass spectrometry (SPME-GC–MS). In total, 417 nonvolatile and 55 volatile substances were detected. The nonvolatile substances were classified into the following 10 categories: organic acids and derivatives (28.9%); organic oxygen compounds (21.9%); lipids and lipid-like molecules (12.6%); organoheterocyclic compounds (9.9%); nucleosides, nucleotides, and analogues (9.4%); benzenoids (7.8%); phenylpropanoids and polyketides (6.1%); organic nitrogen compounds (2.7%); lignans, neolignans, and related compounds (0.5%); and alkaloids and their derivatives (0.3%). The volatile compounds (VOCs) were classified into the following eight categories: organic oxygen compounds (24.1%); organic cyclic compounds (20.4%); organic nitrogen compounds (13%); organic acids and their derivatives (13%); lipids and lipid-like molecules (11.2%); benzenoids (11.1%); hydrocarbons (3.7%); and homogeneous non-metallic compounds (3.7%). Differentially abundant metabolites among the different peanut varieties (ZKH13H vs. CFD, ZKH1H vs. CFD, and ZKH1H vs. ZKH13H) were investigated via multivariate statistical analyses, which identified 213, 204, and 157 nonvolatile differentially abundant metabolites, respectively, and 12, 11, and 10 volatile differentially abundant metabolites, respectively. KEGG metabolic pathway analyses of the differential non-VOCs revealed that the most significant metabolic pathways among ZKH13H vs. CFD, ZKH1H vs. CFD, and ZKH1H vs. ZKH13H were galactose metabolism, purine metabolism, and aminoacyl-tRNA, while the nitrogen metabolism pathway was identified as a significant metabolic pathway for the VOCs. The present findings provide a theoretical foundation for the development and utilization of these three peanut species, as well as for the breeding of new peanut varieties.

## 1. Introduction

The peanut (*Arachis hypogaea* L.) is an important agricultural product with a wide range of uses in human life. The planting area is second only to oilseed rape, ranking second in the world for oilseed crops [1]. Peanuts can be consumed directly as snacks or used for the extraction of edible oil and the production of various food products, such as peanut butter, peanut candy, and fried peanuts. Approximately 45–50% of peanuts produced in China are used for oil extraction, while the rest are used to make peanut-based food [2]. China is one of the world’s largest producers of peanuts, with all regions of the country suitable for cultivation and abundant production; the peanut industry plays an important role in China’s agricultural economy, providing a stable economic source for farmers [3]. With the promotion of modern agricultural technology, China’s peanut industry is also continuously improving its technology and production efficiency. However, in recent years, as the yield of peanuts has increased, the evaluation of peanuts has begun to shift from quantity to quality.

Flavor is one of the most important indicators for evaluating the quality of peanut varieties, and it largely affects consumer preferences and purchase intentions [4]. Aroma and taste play important roles in flavor perception. The chemical composition of the matrix and consequently its structure influence the release and perception of flavor. The peanut’s aroma is derived from various volatile organic compounds produced by the peanut. Taste refers to the ability of the human mouth to evaluate aroma and taste together, forming its flavor. Flavor components include non-volatile and volatile substances, with free amino acids and 50 nucleotides comprising the main nonvolatile flavor materials [5,6]. Certain varieties of peanuts have distinct and heritable flavor characteristics [7]. There are numerous varieties of peanuts available worldwide, each with their own distinct flavor. Therefore, the study of the differences in peanut flavor substances among the different varieties can help to comprehensively understand the genetic mechanism of peanut flavor substances and provide support for their commercial application. As a result, researchers have conducted numerous investigations on the detection of peanut flavor substance composition over the years. In 1968, Brown et al. developed a technique for extracting quantities of peanuts and separating these extracts into fractions of different chemical categories, each with a distinct flavor, and 12 acids were identified [8]. Walradt et al. identified 187 volatiles from roasted peanuts by preparative gas phase separation and GC–MS determination [9]. In 2006, the volatiles from freshly roasted peanuts were evaluated over a 21-day storage period using gas chromatography, chemosensory techniques, and a sensory panel [10]. In recent years, researchers have focused increasingly on volatile flavor compounds. Ultrahigh-performance liquid chromatography-quadrupole time-of-flight mass spectrometry (UHPLC–Q-TOF/MS) and headspace solid-phase microextraction–gas chromatography–mass spectrometry (HS-SPME–GC–MS) are efficient and well-established methods for separating various nonvolatile and volatile flavor substances [11]. These methods have been successfully applied in the detection of flavor substances in oats [12], *Houttuynia cordata* Thunb. [13], corn wine [14], and beef [15]. Metabolomics can be used to elucidate the relationships between the aroma compounds of peanuts and their sensory properties, providing a scientific reference for the regulation of peanut processing and the breeding of new varieties. Klevorn et al. and Zhang et al. used metabolomics to determine changes in the composition of small molecular weight compounds, nonvolatile metabolites, and volatile metabolites in dry roasted peanuts [16,17]. The GC–MS approach has also been used to study lipid changes during the cooking of high-oleic acid peanuts and to evaluate the flavor characteristics of peanut butter pretreated by radio frequency heating, explosion puffing, microwaves, and oven heating [18,19]. The flavor substances of different varieties of peanut butter have also been studied via GC–MS [20]. In previous studies, flavor changes during the processing of roasted peanuts, peanut butter, and peanut oil have been studied using metabolomics techniques [4,21,22,23]. However, the flavor of different varieties of raw peanuts has been less studied. Ding et al. measured the flavor characteristics of ten peanut varieties from China via high-performance liquid chromatography (HPLC) and gas chromatography–mass spectrometry (GC–MS), but they did not include metabolomics [24]. In our study, the volatile and nonvolatile metabolites of three varieties of peanuts were analyzed using flavoromics technology, which can analyze multiple compounds at the same time, including aroma components, flavor components, and other substances that influence flavor, to provide comprehensive information on flavor profiles. Compared to other traditional analytical procedures, very little amounts of chemicals can be identified. This will provide the foundation for a more accurate assessment of the flavor differences between the different varieties of peanuts.

Peanut is the major oil crop in Guangdong Province, China, and it is also the second largest crop, with the planting area ranking third in China, second only to Henan Province and Shandong Province. Zhongkai Hua 1 hao (ZKH1H), Zhongkai Hua 13 hao (ZKH13H), and Chifandou (CFD) are the main peanut varieties in this region. Therefore, these three peanut varieties from Guangdong Province were selected as research objects in this study. HS-SPME–GC–MS and UPLC–Q-TOF/MS were developed for the determination of the VOC and non-VOC differences among the peanut varieties. To provide theoretical guidance for peanut variety selection and processing applications, the differentially abundant metabolites were analyzed among the peanut varieties, and the differential metabolic pathways were enriched to reveal the metabolic variability of the peanut varieties.

Assification of differentially abundant metabolite functions were performed via the Kyoto Encyclopaedia of Genes and Genomes (KEGG) database.

## 2. Results

**Figure 1:**
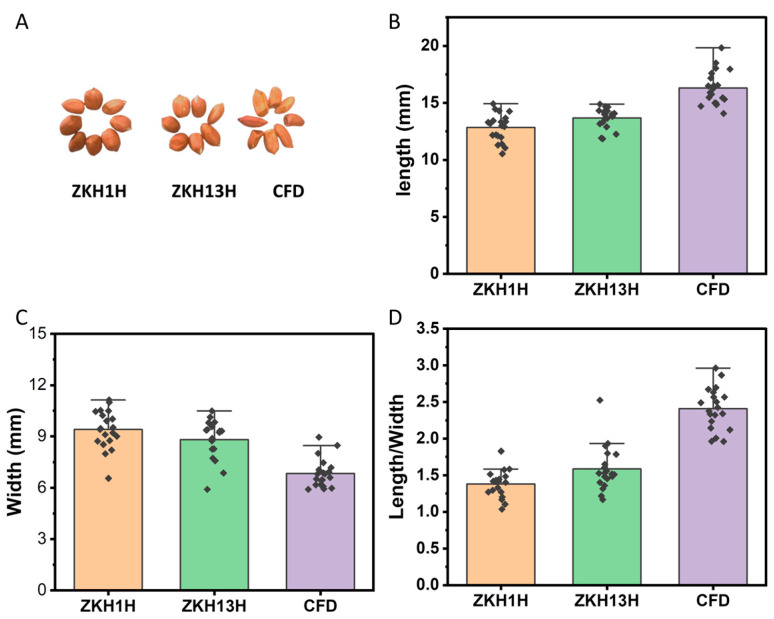
The appearance (**A**), length (**B**), width (**C**), and length/width ratio (**D**) of the three varieties of peanuts. The data are presented as the mean ± standard deviation (SD).

Figure 1 presents the appearance of the three varieties of peanuts, as well as the length, width, and aspect ratio values of the individual peanuts. The mean lengths of the peanut kernels of ZKH1H, ZKH13H, and CFD were 12.84 ± 1.21 mm, 13.67 ± 0.88 mm, and 16.32 ± 1.48 mm, respectively. The length of the CFD variety was significantly greater than those of ZKH1H and ZKH13H by 27.07% and 19.36%, respectively (*p* < 0.001 and *p* < 0.001, respectively). There was no significant difference between the lengths of ZKH1H and ZKH13H (*p* = 0.087) (Figure 1B). In terms of width, ZKH1H had the greatest peanut kernel width, and the CFD variety had the smallest peanut kernel width. However, there was no significant difference in width between ZKH1H and ZKH13H (*p* = 0.195). The width of the CFD variety was significantly less than those of ZKH1H and ZKH13H (*p* < 0.001 and *p* < 0.001, respectively) (Figure 1C). The ratio of length to width is one of the morphological indicators of peanuts, and there were significant differences in the length-width ratios among the three varieties, with the CFD variety having the largest length-width ratio and ZKH1H having the smallest (Figure 1D).

**Figure 2:**
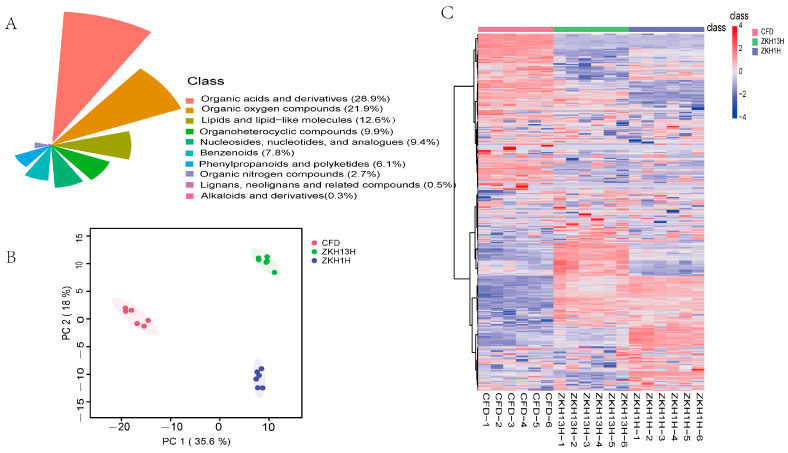
Different main nonvolatile metabolites in the three varieties of peanut samples were identified through a widely targeted metabolic method and method reliability evaluation. (**A**) Classification of the 417 nonvolatile metabolites detected in the three varieties of peanut samples. (**B**) PCA score plot. (**C**) Heatmap analysis.

**Figure 3:**
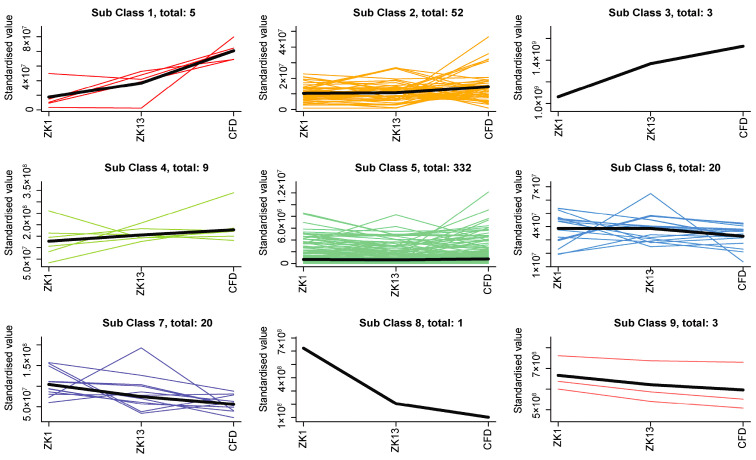
K-means clustering analysis of all nonvolatile metabolites of the three varieties of peanut samples.

In present study, the UPLC–Q-TOF/MS method was used to identify the differences between the nonvolatile metabolites of the three varieties of peanuts; the total ion current (TIC) overlap plots and multipeak detection plots of the peanut samples are shown in Figure S1. The high overlap rates of the TIC curves observed across various peanut samples provide strong evidence of the reproducibility and reliability of the evaluation outcomes. The structures of the nonvolatile metabolites were determined via a comparative analysis with the MWDB database, and 417 nonvolatile metabolites in the following 10 classes were identified from the three peanut species (Figure 2A): organic acids and their derivatives (28.9%); organic oxygen compounds (21.9%); lipids and lipid-like molecules (12.6%); organoheterocyclic compounds (9.9%); nucleosides, nucleotides, and analogues (9.4%); benzenoids (7.8%); phenylpropanoids and polyketides (6.1%); organic nitrogen compounds (2.7%); lignans, neolignans, and related compounds (0.5%); and alkaloids and their derivatives (0.3%). To analyze the differences among the three peanut varieties, the samples were subjected to principal component analysis (Figure 2B), which revealed that the contribution of principal component 1 was 35.6% and that of principal component 2 was 18%. The peanut samples in each group were clustered together within the group, and the intragroup variability was small and highly reproducible. The groups were clearly separated, indicating that the metabolite fractions of different peanut samples were significantly different. After the metabolite data were normalized, cluster analysis was performed on all the samples. There were different clustering patterns among the different species (Figure 2C). Furthermore, K-means plots were utilized to cluster the relative contents of the nonvolatile metabolites obtained from the screening of all subgroups. All the metabolites were classified into nine subclasses, containing 5, 62, 3, 9, 332, 20, 20, 1, and 3 metabolites (Figure 3).

**Figure 4:**
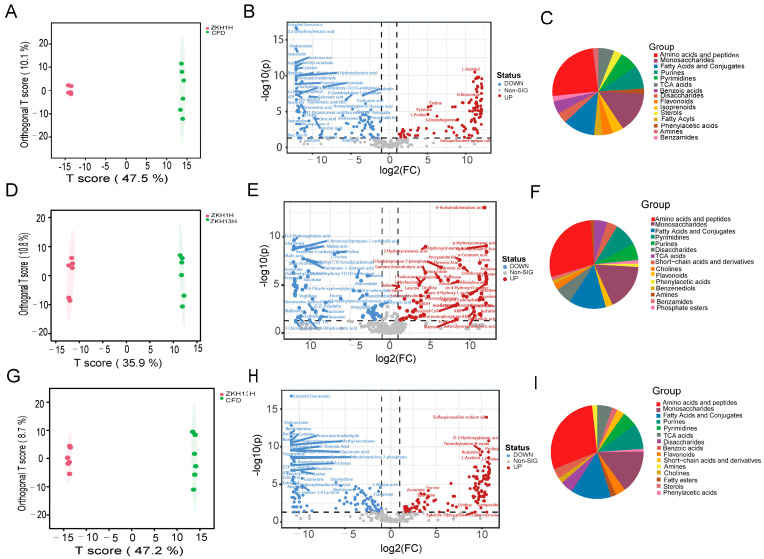
Differential nonvolatile metabolites obtained by comparison of each pair of varieties. Score plots of OPLS-DA pairwise comparisons of differentially abundant metabolites in (**A**) ZKH1H vs. CFD, (**D**) ZKH1H vs. ZKH13H, and (**G**) ZKH13H vs. CFD. Volcano plots showing the differential nonvolatile metabolite expression levels in (**B**) ZKH1H vs. CFD, (**E**) ZKH1H vs. ZKH13H, and (**H**) ZKH13H vs. CFD. Classification of the differential nonvolatile metabolites obtained by comparison of each pair of varieties in (**C**) ZKH1H vs. CFD, (**F**) ZKH1H vs. ZKH13H, and (**I**) ZKH13H vs. CFD.

To gain a deeper insight into the variations in non-VOCs among the three varieties, OPLS-DA was used to further screen out the differential non-VOCs between the pairwise comparisons of ZKH1H, ZKH13H, and CFD (Figure 4). The OPLS-DA model score plot (Figure 4A,D,G) indicated significant separations among the different raw peanuts, suggesting differences in their respective metabolite profiles. The criteria, VIP ≥ 1 and fold change ≥2 or ≤0.5, were utilized to screen for differentially abundant metabolites, which were visualized via volcano plots. In the ZKH1H vs. CFD comparison, 204 differentially abundant metabolites were identified (Figure 4B), of which 105 metabolites were upregulated and 99 were downregulated (Table S1). The differentially abundant metabolites were classified as follows: amino acids and peptides (24.76%); monosaccharides (15.24%); fatty acids and conjugates (11.43%); purines (8.57%); pyrimidines (6.67%); TCA acids (5.71%); benzoic acids (4.76%); disaccharides (3.81%); flavonoids (3.81%); isoprenoids (3.81%); sterols (2.86%); fatty acyls (2.86%); phenylacetic acids (1.90%); amines (1.90%); and benzamides (1.90%) (Figure 4C). In the ZKH1H vs. ZKH13H comparison, 157 significantly different nonvolatile metabolites were detected (Figure 4E), of which 77 were upregulated and 80 were downregulated (Table S2). These compounds were classified as follows: amino acids and peptides (28.40%); monosaccharides (17.28%); fatty acids and conjugates (13.58%); pyrimidines (8.64%); purines (6.17%); disaccharides (6.17%); TCA acids (4.94%); short-chain acids and derivatives (3.70%); cholines (2.47%); flavonoids (2.47%); phenylacetic acids (1.23%); benzenediols (1.23%); amines (1.23%); benzamides (1.23%); and phosphate esters (Figure 4F). In the ZKH13H vs. CFD comparison, 213 differentially abundant metabolites were identified (Figure 4H), of which 116 were upregulated and 97 were downregulated (Table S3). These compounds were classified into 15 categories as follows: amino acids and peptides (29.20%); monosaccharides (15.04%); fatty acids and conjugates (14.16%); purines (8.85%); pyrimidines (5.31%); TCA acids (5.31%); disaccharides (4.42%); benzoic acids (3.54%); flavonoids (3.54%); short-chain acids and derivatives (2.65%); amines (1.77%); cholines (1.77%); fatty esters (1.77%); sterols (1.77%); and phenylacetic acids (0.88%) (Figure 4I).

**Figure 5:**
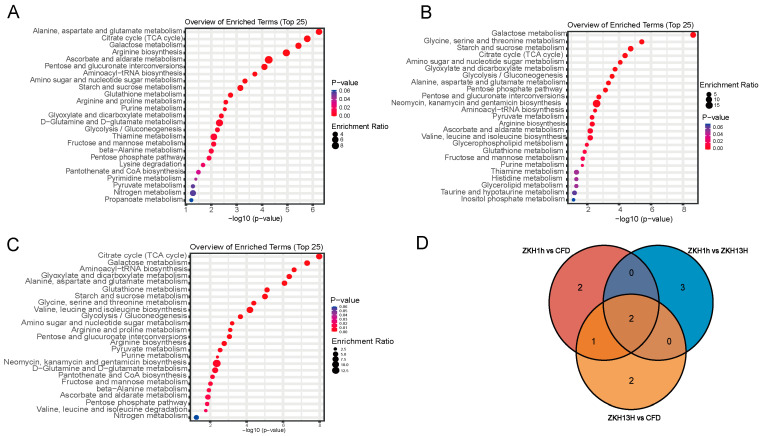
KEGG pathway annotation of the differential nonvolatile metabolites obtained by comparing each of the two groups in (**A**) ZKH1H vs. CFD, (**B**) ZKH1H vs. ZKH13H, and (**C**) ZKH13H vs. CFD. (**D**) Intersection of the 5 pathways with the most significant differences in the 3 comparison groups.

To identify the perturbed biological pathways, KEGG pathway analyses of the differentially abundant metabolites between the pairwise comparisons were performed via the KEGG database, and the top 25 pathways with the most significant differences in each comparison group are shown in Figure 5. In the ZKH1H vs. CFD comparison, differentially abundant metabolites were distributed to a total of 55 metabolic pathways, of which 25 had significant differences. The five most significantly different pathways were as follows: alanine, aspartate, and glutamate metabolism; citric acid cycle (TCA cycle); galactose metabolism; arginine biosynthesis; ascorbic acid; and aldolate metabolism (Figure 5A). In the ZKH1H vs. ZKH13H comparison, all differentially abundant metabolites were distributed into 51 metabolic pathways, of which 42 had significant differences (*p* < 0.05). The five most significant differences were as follows: galactose metabolism; glycine, serine, and threonine metabolism; starch and sucrose metabolism; citric acid cycle (TCA cycle); and amino acid and nucleotide glucose metabolism (Figure 5B). In the ZKH13H and CFD comparison, differentially abundant metabolites were distributed across 55 metabolic pathways, of which 42 pathways presented significant differences (*p* < 0.05). The five most significant differences were as follows: citric acid cycle (TCA cycle); galactose metabolism; aminoacyl-tRNA biosynthesis; glyoxylate and dicarboxylic acid metabolism; and alanine, aspartate, and glutamic acid metabolism (Figure 5C). To identify the metabolic pathways with the most significant effects on the different nonvolatile metabolites, the intersection of the five pathways with the most significant differences in the three comparison groups were obtained, which identified two common metabolic pathways, namely the citrate cycle (TCA cycle) and galactose metabolism (Figure 5D). There were two (arginine biosynthesis; ascorbate and aldarate metabolism), three (glycine, serine and threonine metabolism; starch and sucrose metabolism; and amino sugar and nucleotide sugar metabolism) and two (aminoacyl-tRNA biosynthesis; and glyoxylate and dicarboxylate metabolism) unique metabolic pathways in the ZKH1H vs. CFD, ZKH1Hvs. ZKH13H, and ZKH13H vs. CFD comparison groups, respectively (Figure 5D).

**Figure 6:**
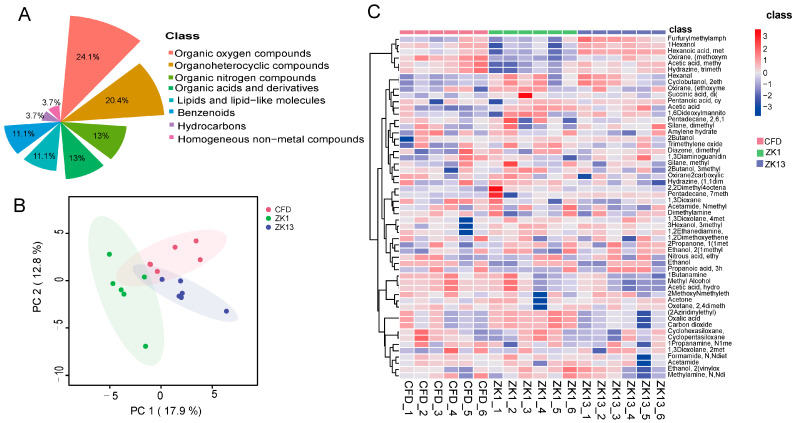
Multivariate statistical analysis of VOCs in the three varieties of peanut samples. (**A**) Classification of the 55 nonvolatile metabolites detected in the three varieties of peanut samples. (**B**) PCA score plot. (**C**) Heatmap analysis.

A total of 55 VOCs were detected in the peanut samples. The majority of these compounds were present in all three samples (Table S4) as follows: organic oxygen compounds (24.1%); organoheterocyclic compounds (20.4%); organic nitrogen compounds (13%); organic acids and their derivatives (13%); lipids and lipid-like molecules (11.1%); benzenoids (11.1%); hydrocarbons (3.7%); and homogeneous non-metallic compounds (3.7%) (Figure 6A). To explore the differences between the metabolites of the peanut varieties, the sample data were subjected to a principal component analysis (PCA) (Figure 6B). The data points of the three groups of peanuts were clearly distinguished on the score plot. After normalization of the metabolite data, cluster analysis was performed on all the samples. Cluster analysis classifies metabolites with the same characteristics into groups and identifies the degree of variation in the levels of metabolites with the same characteristics within a group. The results revealed that there were different clustering patterns among the different varieties (Figure 6C).

**Figure 7:**
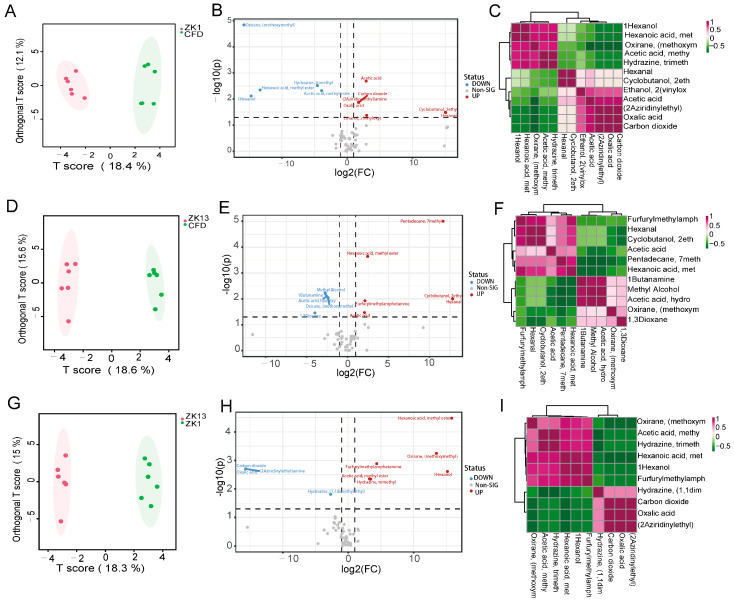
Differential VOCs obtained by comparison of each pair of varieties. Score plots of OPLS-DA pairwise comparisons of differentially abundant metabolites in (**A**) ZKH1H vs. CFD, (**D**) ZKH1H vs. ZKH13H, and (**G**) ZKH13H vs. CFD. Volcano plots showing the differential nonvolatile metabolite expression levels in (**B**) ZKH1H vs. CFD, (**E**) ZKH1H vs. ZKH13H, and (**H**) ZKH13H vs. CFD. Correlation analysis of differential volatile metabolites in (**C**) ZKH1H vs. CFD, (**F**) ZKH1H vs. ZKH13H, and (**I**) ZKH13H vs. CFD.

OPLS-DA also revealed significant differences in the VOC pairwise comparisons among the three varieties of peanuts, with VIP ≥ 1.0, FC ≥ 2/≤0.5, and *p* < 0.05 indicating significant differences in the VOCs (Figure 7A,D,G). Because metabolite molecules are strongly correlated with each other, it is possible to identify metabolite molecules that are more highly correlated in a sample via a correlation analysis of the levels of metabolite molecules in different samples. Therefore, a correlation analysis of the differentially abundant metabolites obtained from different comparison groups was performed, and the heatmap of the correlation results is shown in Figure 7C,F,I. In the ZKH1H vs. CFD comparison, 12 differential VOCs were identified (Figure 5B), of which 7 VOCs were upregulated and 5 were downregulated, and the differentially abundant metabolites are shown in Table S5. In the ZKH13H vs. CFD comparison, 11 differential VOCs were identified (Figure 7E), of which 6 VOCs were upregulated and 5 were downregulated, and the differentially abundant metabolites are shown in Table S6. Finally, in the ZKH1H vs. ZKH13H comparison, ten differential VOCs were obtained, of which six were upregulated and four were downregulated (Table S7). This suggests that these compounds may significantly contribute to the fragrance characteristics of a peanut’s aroma.

**Figure 8:**
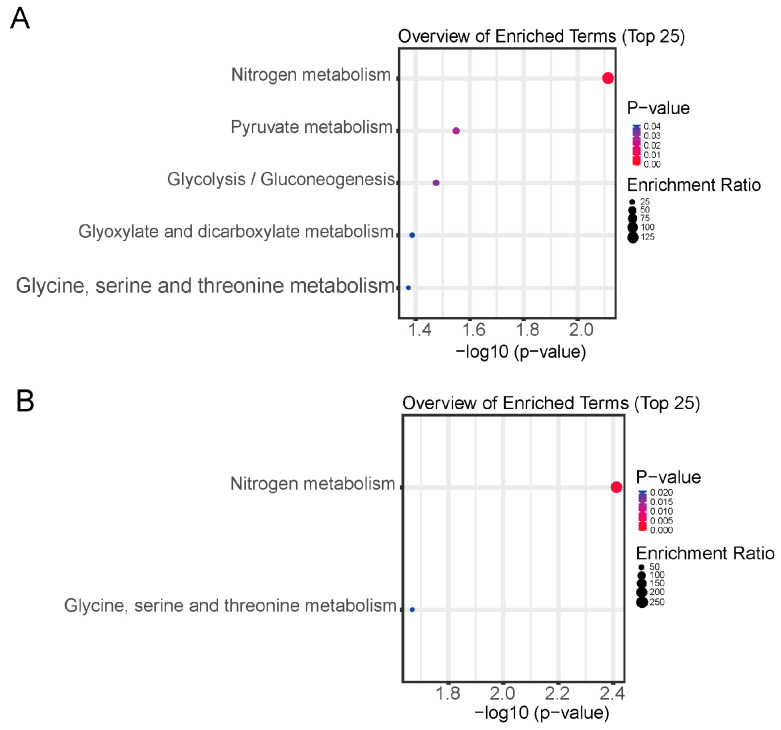
Differential VOCs from GC-MS were enriched in distinct KEGG pathways by comparing each of the two groups. (**A**) ZKH1H vs. CFD, (**B**) ZKH1H vs. ZKH13H.

Also, KEGG pathway analyses of the differentially abundant VOCs between the pairwise comparisons were also performed via the KEGG database, and the significant differences in each comparison group are shown in Figure 8. In the ZKH1H vs. CFD comparison, the following five metabolic pathways were significantly enriched (*p* < 0. 05): nitrogen metabolism; pyruvate metabolism; glycolysis/gluconeogenesis; glyoxylate and dicarboxylate metabolism; and glycine, serine and threonine metabolism (Figure 8A). There were no significant differences in metabolic pathways in the ZKH13H vs. CFD comparison. In the ZKH1H vs. ZKH13H comparison, the following two metabolic pathways were significantly enriched: nitrogen metabolism and glycine; and serine and threonine metabolism.

## 3. Discussion

Peanuts, a commercial oilseed crop, provide high-quality vegetable oil and a source of nutrients for the human diet. The metabolites of peanuts have attracted much attention in academic research and peanut production [17,25,26,27,28,29,30]. There are numerous peanut varieties, and the quality of peanut varieties promoted for use in current production varies greatly among the varieties not only in nutritional quality but also in sensory quality. In the present study, three varieties of peanuts (ZKH1H, ZKH13H, and CFD) with different morphologies (Figure 1A) were selected to reveal the volatile and nonvolatile metabolites via widely targeted metabolomics. ZKH varieties are the main peanut varieties grown in Guangdong, China. The ZKH1H and ZKH13H varieties have been popularized and planted in larger areas in recent years. The CFD varieties of peanuts are the peanut varieties that are traditionally grown in Heyuan city, Guangdong. A visual comparison indicated that the peanut grains of the CFD variety were longer and finer (Figure 1B–D). Peanut flavor is the most important characteristic influencing consumer purchase intentions, and this characteristic is highly dependent on the cultivar [30,31]. The present study investigated volatile and nonvolatile substances in three peanut varieties via flavoromics techniques, which identified 417 non-VOCs and 55 VOCs in the peanut varieties. Furthermore, OPLS-DA was used to screen out the differential nonvolatile metabolites among the pairwise comparisons of ZKH1H, ZKH13H, and CFD. The KEGG pathways of the differentially expressed nonvolatile metabolites and volatile organic compounds (VOCs) were annotated. Two core metabolic pathways affecting the nonvolatile metabolites and one core metabolic pathway affecting the volatile metabolites were identified after obtaining the intersection. This study provides a basis for the study of volatile and nonvolatile compounds in different varieties of peanuts.

Peanut seeds are rich in high-quality fatty proteins and contain eight essential amino acids [32], which are known to be the precursors of peanut flavor substances [33]. In the present study, the core differences between the nonvolatile metabolites in the comparisons accounted for the largest proportion of amino acids and peptides (Figure 4C,F,I). The following metabolic pathways unique to the ZKH1H vs. CFD, ZKH1H vs. ZKH13H, and ZKH13H vs. CFD comparisons groups were all metabolic pathways related to amino acid synthesis: arginine biosynthesis; ascorbate and aldarate metabolism; glycine, serine and threonine metabolism; starch and sucrose metabolism; amino sugar and nucleotide sugar metabolism; aminoacyl-tRNA biosynthesis; and glyoxylate and dicarboxylate metabolism (Figure 5). Therefore, these findings suggest that the nonvolatile metabolites of these three varieties of peanuts are caused mainly by differences in amino acid metabolism. Afolabi et al. discovered that different peanut genotypes result in different amino acid contents, which is an important factor in the formation of peanut flavor [34]. In addition, each amino acid type and amino acid content in different varieties of peanuts contribute differently to the development of flavor [35,36]. Among them, aspartic acid and glutamic acid directly affect the typical flavor of peanuts [37]. Therefore, evaluating the nutritional value of protein amino acids in different peanut varieties can provide a reference basis for peanut variety selection, development, and utilization. In the present study, differences in the amino acid contents of the three varieties of peanuts may have contributed to their different flavors.

Food flavor is the aroma of various volatile organic compounds produced in food. The peanut aroma is composed of a variety of volatile compounds, including pyrazines, furans, pyridines, aldehydes, ketones, acids, hydrocarbons, aromatics, and other compounds, with different volatile compounds having different aromas. In the present study, there were 12, 11, and 10 differential VOCs in the ZKH1H vs. CFD, ZKH13H vs. CFD, and ZKH1H vs. ZKH13H comparisons, respectively (Figure 7). Oxirane (methoxymethyl) and methyl caproate were different substances common to each group. Methyl caproate has a fresh roasted and vanilla odor, and it is commonly used in food [38], cosmetics [39], and industrial applications [40]. Thus, methyl caproate may have affected the flavor differences among the varieties.

In addition to variety, the growing methods, environmental conditions, seed maturity, and handling methods, and storage methods may contribute to variations in peanut flavor [41]. Six peanut seeds from different locations from the Eastern Mediterranean Agricultural Research Institute have been used to determine sensory attributes and fatty acid composition [42]. Sultan and Aysehanim presented the most liked peanut aroma, whereas NC7 presented the highest crispness score. Gazipasa and Aysehantm had good acceptability for consumers in terms of color and peanut flavor. Climate conditions, especially high temperatures and low amounts of rain during the seed-filling period, affected the fatty acid composition. In the present study, three varieties of peanuts were collected from the same location under consistent growing conditions. Therefore, differences in the flavor of the three peanut varieties should be attributed to the different genotypes. The primary goal of many peanut breeding programs is to improve agronomic performance over existing cultivars. However, it is critical that the desirable sensory characteristics of peanut varieties are preserved during this process.

Peanuts are utilized not only as fresh food but also in the production of roasted peanuts, peanut oil, peanut butter, and other industrialized commodities, where flavor has a significant impact on economic value. Gong et al. studied the organoleptic quality, physicochemical quality, and stability of 18 raw peanut material characteristics and the preparation of peanut butter, and the results showed that there were differences in the raw material characteristics of different varieties of peanuts [43]. In order to determine the volatile compounds associated with the organoleptic properties of peanut varieties in relation to the flavor of roasted peanuts, Gama and Adhikari evaluated the organoleptic properties of six dominant Malawi peanut varieties and the flavor of roasted peanuts, and they found not only that pyrazines and furans were the dominant volatile compounds but also that there was a significant difference in the concentration of each of them [44]. Raw agricultural commodities, such as peanuts, are processed into value-added food products where the amount of active metabolism occurring is diminished due to the destruction of enzymes during processing. Therefore, the interpretation of the metabolomic measurements of processed food products must discuss how the compositions of low molecular weight compounds change as a result of processing.

The present study investigated the flavor characteristics of fresh peanuts to lay the groundwork for peanut processing and variety selection. However, the direct consumption of peanuts accounts for a small portion of their use. Future research on the flavor of roasted peanuts over time will help us to understand how the identified compounds change during storage. Furthermore, analyzing the flavor of the peanuts via consumer and descriptive panels may reveal information about how the compounds influence the roasted flavor and which peanut is preferred by consumers if there is a significant difference.

## 4. Materials and Methods

### 4.1. Samples

Three different varieties of peanuts were collected from Tuo town, Longchuan County, Heyuan city, Guangdong Province, China. The peanuts were dried naturally and then deshelled. All samples were stored at −20 °C until further analysis. The samples were sealed and thawed at room temperature for 1 h before the experiment.

### 4.2. Chemicals

Methanol (GC grade, purity ≥ 95%) and acetonitrile (GC grade, purity ≥ 95%) were purchased from Honeywell International Inc. (Morristown, NJ, USA). Ammonium acetate (GC grade, purity ≥ 95%) was purchased from Sigma-Aldrich (St. Louis, MO, USA). The n-alkane solution (C6-C24) was obtained from Waters (Milford, MA, USA).

### 4.3. Measurements of Peanut Kernel Length and Width

The length and width of the peanut kernels were measured via electronic vernier calipers (Shanghai Measuring Instrument Co., Shanghai, China).

### 4.4. Analysis of Nonvolatile Compounds by HPLC-MS

#### 4.4.1. Sample Pre-Treatment

The sample pretreatment was performed with reference to the previous literature by Kefale et al. [45]. After thoroughly combining the peanut samples, a grinder was used to turn them into powder, and 100 mg of sample was weighed. Next, 1000 μL of extraction solution (methanol-acetonitrile-water = 2:2:1, *v*/*v*) was added and mixed by shaking. The sample was ultrasonicated in an ice-water bath for 10 min and snap frozen in liquid nitrogen for 1 min. This process was repeated three times. The sample was stored at -20 °C for 1 h and then centrifuged at 13,000 rpm for 15 min at 4 °C. The supernatant was dried with a nitrogen blower. Next, 100 μL of acetonitrile–water = 1:1 (*v*/*v*) was added to redissolve the mixture. The mixture was shaken for 30 s, sonicated in an ice-water bath for 10 min, and centrifuged at 13,000 rpm for 15 min at 4 °C. The supernatant was removed, and the sample was prepared for detection.

#### 4.4.2. Detection of Non-VOCs

The extracted samples were subjected to analysis using an UPLC-ESI-MS/MS system comprising a Shimadzu 30A UPLC (Kyoto, Japan) and an Applied Biosystems 5600 Q-TOF/6500 Q-TRAP Mass Spectrometer (AB sciex, Boston, MA, USA). Liquid chromatography tandem time-of-flight mass spectrometry (5600 Q-TOF) was utilized to determine the accurate molecular weights of each metabolite’s standard. It provided detailed measurements of the different peak integrations and secondary fragmentation information, which were crucial for identifying and quantifying the substances. Liquid chromatography tandem quadrupole mass spectrometry (6500 Q-TRAP) operates based on the standard metabolite information, employing multiple reaction monitoring (MRM) to conduct simultaneous detection. This method enhances the precision and reliability of the quantification processes by targeting specific metabolite transitions, thereby ensuring high specificity in detection. The detection and analysis methods were optimized following the protocol previously described by Kefale et al. [26] and carried out by Verygenome Technology Co., Ltd. (Guangzhou, China). Both instruments utilized the same chromatographic conditions and electrospray ionization (ESI) source.

Chromatographic conditions: A Waters UPLC BEH Amide column (1.7 μm, 2.1 × 100 mm) was used to analyze the non-VOCs. Solvent A comprised ultrapure water with 25 mM CH_3_COONH_4_ and 25 mM NH_4_OH, and solvent B comprised chromatographically pure acetonitrile. The gradient elution procedure was as follows: starting at 0 minutes with 90% solvent B; from 0 to 9 min, the proportion of solvent B is reduced to 40%; at 9 to 9.05 minutes, it returns to 90% B; and maintains 90% B from 9.05 to 11.5 min. The flow rate was 0.3 mL/min. The injection volume was 5 μL, and the column temperature was 55 ℃. The effluent was alternatively directed to the ESI–Q-TOF–MS system.

Mass spectrum conditions: The ESI source operation parameters were as follows: the source temperature was 600 °C; the ion spray voltage was 5500 V in positive ion mode and −4500 V in negative ion mode; the curtain gas pressure was 20 psi; and the nebulizer and auxiliary gas pressure was 60 psi. Scanning was performed via multiple reaction monitoring (MRM). With the collision gas (nitrogen) adjusted to the medium during the multiple reaction monitoring mode (MRM) tests, QQQ scans were collected. The raw data were converted to the ‘.mzXML’ format via ProteoWizard, and peak alignment, retention time correction, and peak area extraction were performed via the XCMS program (http://enigma.lbl.gov/xcms-online/, accessed on 17 September 2024). Metabolite structures were accurately matched (<25 ppm) using primary and secondary spectra obtained from a self-constructed database (VGDB) and public databases. The data were normalized, statistically analyzed and then plotted via R software (version R-4.3.2).

Quantitative and qualitative analyses of non-VOCs: To accurately identify the metabolites, the retention time, fragmentation pattern, and precise *m/z* value were compared to standards in the self-compiled metabolite database (VGTB, Guangzhou, China). The quantitative analysis was conducted based on the signal intensity of metabolites derived from various ions. MS signals, including isotopes, K^+^, Na^+^, and NH_4_^+^ adduct ions, were eliminated. Chromatographic peaks were meticulously merged and modified with the MultiaQuant software (version 3.0.3). The area beneath each chromatographic peak indicated the relative concentration of the relevant chemical.

### 4.5. Analysis of Volatile Compounds by Solid-Phase Microextraction-Gas Chromatography-Mass Spectrometry (SPME-GC-MS)

#### 4.5.1. Sample Preparation

The peanut powder (3.0 g) was promptly transferred to a 20 mL headspace vial (Agilent, Palo Alto, CA, USA), followed by the addition of 2 mL of saturated carbinol solution and 10 μL of styrene-d8 (5 μg/mL) as an internal standard. Automated solid-phase microextraction (SPME) was employed for sample extraction; each vial was held at 60 °C for 30 min, after which a 120 μm DVB/CWR/PDMS fibre (Agilent, CA, USA) was exposed to the headspace of the sample at 260 °C for 5 min. Each sample was subjected to this procedure four times.

#### 4.5.2. Detection of VOCs 

VOC detection was performed via gas chromatography-mass spectrometry (GC–MS) using a Model 7890B GC system (Agilent, CA, USA) and a PEGASUS® BT GC–TOFMS system (Leco, Chicago, IL, USA).

Chromatographic condition: A 19091S-433 HP-5MS capillary column (30 m × 0.25 mm × 0.25 μm) with high-purity helium (99.999%) and a linear velocity (1.0 mL/min) was used. The injection mode consisted of a nonsplit injection, and the temperatures of the injector and detector were set at 270 °C and 305 °C, respectively. The oven was initially set to 40 °C. The temperature of the samples was then gradually raised at a rate of 15 °C/min to reach 125 °C. The temperature was subsequently increased at a rate of 5 °C/min and then at a rate of 10 °C/min until it reached 210 °C. Finally, to achieve a final temperature of 305 °C, the oven temperature was increased at a rapid rate of 20 °C/min. This peak temperature was maintained for 5 min. The VOCs were also detected by Verygenome Technology Co., Ltd., Guangzhou, China.

Mass spectrum conditions: The mass spectra were acquired in the electron impact (EI) ionization mode with an energy of 70 eV. The temperatures of the ion source, quadrupole mass detector, and transfer line were set to 240 °C, 160 °C, and 270 °C, respectively. The mass scanning range was 50–600 *m*/*z*, with a 5 min delay to start acquisition. The scanning speed, frequency, and step size were 1562 U/s, 2.7 (number of scans/s), and 0.1 *m*/*z*, respectively. The identification and quantification analysis were performed using the selected ion monitoring (SIM) mode.

Quantitative and qualitative analyses of VOCs: Peak deconvolution was performed on the original file via Qualitative Workflows software (version B.08.00), and peaks were identified by searching NIS 17.0. The peaks were then aligned and quantified via Qualitative Workflow B.08.00 quantitative analysis software and corrected according to the internal standard response. The Qualitative Workflow B.08.00 software setup parameters were as follows: compound discovery, find by molecular features; peak filtration, peak height ≥600 counts; compound identification, asymmetry (*m*/*z*) of −0.3 Da + 0.7 Da; smallest match (*m*/*z*), 30 Da; abundance ratio contrast uncertainty, 40%; and score (rev), 60.00. VOCs were semi-quantified and analyzed based on the peak areas of the internal standard using Equation (1) as follows:

(1) $$Relative\;content\;(\mu g/g)=\frac{The\;content\;of\;IS\;(\mu g)\times \frac{Peak\;area\;of\;target\;comounds}{Peak\;area\;of\;IS}}{Amount\;of\;peanut\;(g)}$$

### 4.6. Statistical Analysis

All tests were performed in triplicate, and the results are expressed as the means ± standard deviations. Statistical analyses were conducted via ANOVA and Tukey’s test in IBM SPSS version 24 (IBM SPSS, Armonk, NY, USA) to determine the significant differences between the different samples. The bar graphs were plotted via Origin software version 2021 (OriginLab Corporation, Northampton, MA, USA). Multivariate statistical analysis techniques were used to process the metabolomic data. Principal component analysis (PCA) was performed using the statistical functions in R (https://www.r-project.org, accessed on 17 September 2024). For the identification of differentially abundant metabolites between the two cultivars, the selection criteria were established with variables importance in project (VIP) scores ≥ 1 and a fold change ≥2 or ≤0.5. VIP values were extracted from the partial least squares discriminant analysis (PLS-DA) results, which were plotted as a volcano plot via the MetaboAnalyst R package (version 4.0), Heatmap analysis was conducted via the Metabo Analyst R package. A hierarchical cluster analysis (HCA) of the metabolites was performed via the ComplexHeatmap R package (version 2.15.4), and the results were displayed as heatmaps with dendrograms.

## 5. Conclusions

The present study investigated the volatile and nonvolatile profiles of different varieties of raw peanuts. A total of 417 non-VOC_S_ were detected in the three varieties of raw peanuts via UPLC-MS. Amino acids, peptides, monosaccharides, fatty acids, and conjugates comprised more than 40% of the differences in non-VOC content, which may lead to differences in the amounts of esters and contribute to the sweetness and overall flavor of raw peanuts. Moreover, 55 VOCs were detected via HS-SPME-GC-MS. Oxirane (methoxymethyl) and methyl caproate were differentially substances common to each group. The KEGG pathways of the differential nonvolatile metabolites and VOCs were annotated, and two core metabolic pathways (TCA cycle and galactose metabolism) affecting the nonvolatile metabolites and one affecting the volatile metabolites (nitrogen metabolism) were identified via the intersections. In conclusion, the present study performed metabolomic analyses of the volatile and nonvolatile metabolites of three peanut varieties, and abundant differentially abundant metabolites were screened. Metabolic pathway enrichment analyses of differentially abundant metabolites were performed, providing a theoretical basis for peanut variety selection, peanut breeding, processing, and the research and development of byproducts.

## Data Availability

The original contributions presented in the study are included in the article/Supplementary Materials, further inquiries can be directed to the corresponding authors.

## Supplementary Materials

The following supporting information can be downloaded at: https://www.mdpi.com/article/10.3390/molecules29225230/s1, Figure S1: LC-MS Overlay display of total ion current (TIC) and multi-peak detection plots for peanut samples; Table S1: The 204 differential nonvolatile metabolites identified in the ZKH1H vs. CFD comparison, of which 105 metabolites were upregulated and 99 were down-regulated; Table S2: The 157 significantly different nonvolatile metab-olites were detected In the ZKH1H vs. ZKH13H comparison, of which 77 were upregulated and 80 were down regulated; Table S3: The 213 differential nonvolatile metabolites identified in the ZKH13H vs. CFD comparison, of which 116 metabolites were upregulated and 97 were down-regulated. Table S4: The total of 55 VOCs were detected in the peanut samples; Table S5. 12 differential VOCs were identified In the ZKH1H vs. CFD comparison, of which 7 VOCs were upregulated and 5 were downregulated. Table S6: 11 differential VOCs were identified in the ZKH13H vs. CFD comparison, of which 6 VOCs were upregulated and 5 were downregulated; Table S7: 10 differential VOCs were identified in the ZKH13H vs. CFD comparison, of which 6 VOCs were upregulated and 4 were downregulated.
