# Supplementary material for: Widely Targeted Metabolomics Method Reveals Differences in Volatile and Nonvolatile Metabolites in Three Different Varieties of Raw Peanut by GC–MS and HPLC–MS
Source: Molecules. 2024 Nov 5;29(22):5230. doi: 10.3390/molecules29225230 (PMC11596408; doi:10.3390/molecules29225230)
Supplement: Supplementary file 1 [file molecules-29-05230-s001.zip › molecules-3240450-supplementary/Supplementary Files/Fig.S1.pdf]

Widely targeted metabolomics method reveals differences in volatile and nonvolatile metabolites in three different varieties of raw peanut by GC-MS and HPLC-MS

Jiantao Fu[1, 2], Yuxing An[1, 2], Dao Yao[3], Lijun Chen[1, 2,] Liwen Zhou[3], Dachun Shen[1, 2], Sixing Dai[1, 2], Yinglin Lu[1, 2]*, Donglei Sun[1, 2]*

1. Institute of Nanfan & Seed Industry, Guangdong Academy of Sciences, Guangzhou, Guangdong, 510316, China

2. Guangdong Province Pesticide-fertilizer Technology Research Center, Guangzhou, Guangdong, 510316, China

3. Heyuan Provincial Academy of Sciences Research Institute, Guangdong Academy of Sciences, GDAS, Heyuan, 517001, China

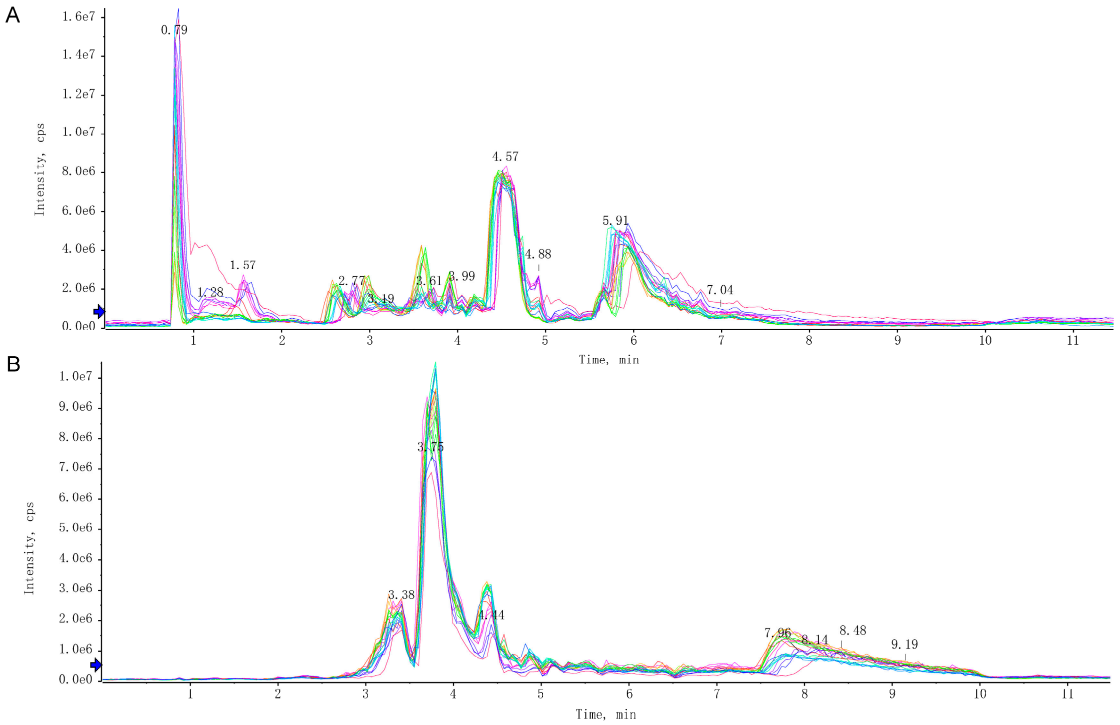

Figure. S1 LC-MS Overlay display of total ion current (TIC) and multi-peak detection plots for peanut samples. A: Positive Ions, B: Negative Ions.
